# First Experience in Small Incision Lenticule Extraction with the Femto LDV Z8 and Lenticule Evaluation Using Scanning Electron Microscopy

**DOI:** 10.1155/2020/6751826

**Published:** 2020-09-26

**Authors:** Mengmeng Wang, Fengju Zhang, Christine Carole Copruz, Longhui Han

**Affiliations:** ^1^Hebei Ophthalmology Key Lab, Hebei Eye Hospital, Xingtai 054000, Hebei Province, China; ^2^Department of Biomedical Engineering, Tufts University, Medford, MA 02155, USA; ^3^Beijing Tongren Eye Center, Beijing Tongren Hospital, Capital Medical University, Beijing Ohthalmology and Visual Sciences Key Lab, Beijing 100730, China; ^4^Ifugao State University Eye Center, Alfonso Lista, Ifugao 3608, Philippines

## Abstract

**Purpose:**

To evaluate the specifications and technique properties of the new Femto LDV Z8 in creating intrastromal refractive lenticules during small incision lenticule extraction (SMILE).

**Methods:**

Six enucleated porcine eyeballs were equally divided into two groups (Femto LDV Z8 or VisuMax) and were randomly assigned to three experienced refractive surgeons who performed SMILE on each group. Five intraoperative time parameters and surgeons' satisfaction on the surgical procedure were compared between two groups. Postoperatively, the roughness of the lenticule surfaces and the irregularity of edges were observed by scanning electron microscopy (SEM) and were also compared between the two groups.

**Results:**

Longer time on suction peak pressure, total laser application, and total surgery were spent in the Femto LDV Z8 group as compared with the VisuMax group. The Femto LDV Z8 group applied OCT scanning and offsetting before performing the laser procedure, which expended more time for these crucial steps. The widest range of surgeons' satisfaction scores was found in the step of lenticule interface identification of the Femto LDV Z8 group. The roughness scores of the anterior and posterior lenticule surfaces were statistically less in the Femto LDV Z8 group than in the VisuMax group (anterior, ×180, *p*=0.039; anterior, ×250, *p*=0.337; posterior, ×180, *p*=0.006; and posterior, ×250, *p*=0.007).

**Conclusions:**

Femto LDV Z8 showed promising performances as a novel SMILE equipment for the correction of myopia. It has special and unique features for SMILE procedures, which need more learning and researching processes. With its low-energy high-frequency nJ-level laser system, the Femto LDV Z8 provided smoother lenticule surface than VisuMax.

## 1. Introduction

Small incision lenticule extraction (SMILE) is a femtosecond laser procedure which was first performed using the VisuMax Laser System (Carl Zeiss Meditec AG, Jena, Germany). [1, 2] This no-flap surgery has shown promising long-term outcomes for the correction of myopic refractive errors similar to those of laser in-situ keratomileusis (LASIK) and photorefractive keratectomy (PRK). [3–5] The Femto LDV Z8 (Ziemer Ophthalmic Systems AG, Port, Switzerland) is a high-frequency femtosecond laser system with a three-dimensional cutting mode. Studies by Pajic et al. [6] and Ebner et al. [7] documented that Femto LDV Z8 usually enables surgeons to perform customized flaps with adjustable self-sealing flap edges and hinges for femtosecond-LASIK procedures. It also enables the spectrum of intrastromal tunnels for intracorneal rings (ICR), intrastromal pockets (ISP) for presbyopic inlay implantation, and intrastromal dissection for lamellar and penetrating keratoplasties, as well as capsulotomy and lens fragmentation for cataract surgery. Recently, an uncommercial mode of Femto LDV Z8 provides a new function on creating lenticules for SMILE. In the current study, the specifications and technique properties of Femto LDV Z8 are evaluated and compared with VisuMax.

## 2. Methods

Since Femto LDV Z8 has not yet acquired approval from the China Food and Drug Administration for commercial and patient use, enucleated porcine eyeballs were utilized in lieu of human eyes for this study. Six enucleated porcine eyeballs were retrieved from local abattoirs within five hours postmortem. These eyeballs, which were numbered from Eye 1 to Eye 6, then were divided into 2 groups (Femto LDV Z8 group and VisuMax group) before delegating to three experienced refractive surgeons. Eyes 1, 2, and 3 were assigned to the Femto LDV Z8 group, while Eyes 4, 5, and 6 were assigned to the VisuMax group. Small incision lenticule extraction was performed accordingly in the following order: Dr. W for Eye 1 and Eye 4, Dr. L for Eye 2 and Eye 5, and Dr. Z for Eye 3 and Eye 6. A Femto LDV Z8 model machine from a medical exhibition in China and a VisuMax Laser commercial machine from Hebei Eye Hospital, China, were used for this study. The same treatment parameters were set on the two laser platforms preoperatively. These included cap thickness, refractive correction, lenticule diameter (optical zone), and lenticule sidecut angle. All the surgeons in this study had more than 10 years of refractive surgical experience, each of them having more than 1000 cases of FS-Lasik operations using Femto LDV Z6 and more than 500 cases of SMILE operations using VisuMax.

**Figure 1:**
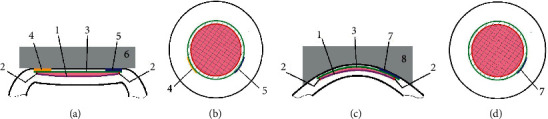
Incision geometry of the SMILE procedures by the two femtosecond laser platforms. (a), (b) The geometry of incisions which were created by the Femto LDV Z8; (c), (d) The geometry of incisions which were created by the VisuMax. 1, incision for cutting the posterior surface of the lenticule; 2, lenticule sidecut; 3, incision for cutting the anterior surface of the lenticule; 4, one of two incisions for opening cap by Femto LDV Z8 (usually used for identifying one side of the lenticule surface); 5, the other of two incisions for opening cap by Femto LDV Z8 (usually used for identifying the other side of the lenticule surface); 6, the flat contact glass of suction system in Femto LDV Z8; 7, the only incision for opening cap by VisuMax (usually used for identifying the two sides of lenticule surfaces ); 8, the curved contact glass of suction system in VisuMax. Pink areas indicate the lenticules after SMILE surgeries.

The machine specifications of the two femtosecond laser platforms are shown in Table 1 and Figure 1. Both the Femto LDV Z8 and VisuMax featured standard techniques of SMILE such as docking, femtosecond laser application, lenticule dissection, and extraction. [8] Nonetheless, there are still subtle differences between the two platforms. The centration of the target lenticule, for example, is based on high resolution OCT images in Femto LDV Z8, but is seen as real-time video in VisuMax; if the centration of the docking does not satisfy the surgeon, quantizable offsetting can be performed without releasing the suction via Femto LDV Z8, but not by VisuMax; Another difference is that two cap opening incisions were created by the Femto LDV Z8, but only one by VisuMax.

In the current study, the differences in suction peak pressure time, OCT scanning time, offsetting time, total laser application time, and total surgery time were compared between the two laser platforms during the surgical procedures of the 6 eyes. After the completion of each procedure, each surgeon's satisfaction was scored using a self-10-point scale based on an evaluating method for a general surgical study. [9] The parameters assessed included cup incision opening, lenticule interface identification, lenticule separation, lenticule removal, and total surgical time.

All lenticules were removed and divided into halves by microscopic scissors and were prepared for scanning electron microscopy (SEM). The anterior surface, posterior surface, and lenticule edge of each of the lenticules were examined at ×180 and ×250 magnification using a Hitachi S–3500 N Scanning Electron Microscope (Hitachi, Ltd., Tokyo, 100–8280, Japan), and images were taken. The SEM images were handed over to three observers in a randomized order to evaluate the roughness of anterior and posterior lenticule surfaces and the irregularity of lenticule edge. The subjective scoring is carried on as a self-10-point scale from 1 for “smoothest/most regular” to 10 for “roughest/most irregular” according to methods used by previous studies. [10, 11].

The statistical package IBM SPSS Statistics 20 (IBM, Armonk, New York, USA) was used in the current study. Data were expressed as mean–standard deviation. The descriptive statistical analysis was performed for comparing the differences of the two laser platforms on suction peak pressure time, OCT scanning time, offsetting time, and total laser application time, as well as the surgeon's satisfaction during the surgical processes. Score data of lenticule surface roughness and edge irregularity were compared between the two laser platform groups using a Mann–Whitney nonparametric test. *p* < 0.05 was considered statistically significant.

## 3. Results

Each laser platform was successfully used for three complete SMILE procedures without complications.

Table 2 shows the surgeons' satisfaction scores on the four general steps of SMILE using the two femtosecond laser platforms. In terms of ease and satisfaction, each surgeon had his own technique preference in the surgical processes. The widest range of satisfaction as rated by the surgeons was found in the ease of lenticule interface definition of the Femto LDV Z8 group, which was from 2 to 6. A narrowest range was in the lenticule removal step for both groups, which was from 1 to 2.

Table 3 shows the time duration of five surgical processes per one eye treatment. In terms of equipment specifications, Femto LDV Z8 has novel features of OCT scanning and offsetting before performing the laser procedure, with mean averages of 12.6 ± 0.5 seconds and 7.0 ± 2.5 seconds, respectively. Statistical comparison between the two laser platforms could not be performed due to a small number of population in this study; nonetheless, an obvious trend of longer duration in suction peak pressure, total laser application, and total surgical time can be observed with the Femto LDV Z8 group (8.3 ± 0.2 seconds, 51.0 ± 1.1 seconds, and 265.1 ± 11.3 seconds, respectively) as compared with the VisuMax group (2.0 ± 0.1 seconds, 21.4 ± 1.0 seconds, and 173.3 ± 7.6 seconds, respectively).

**Figure 2:**
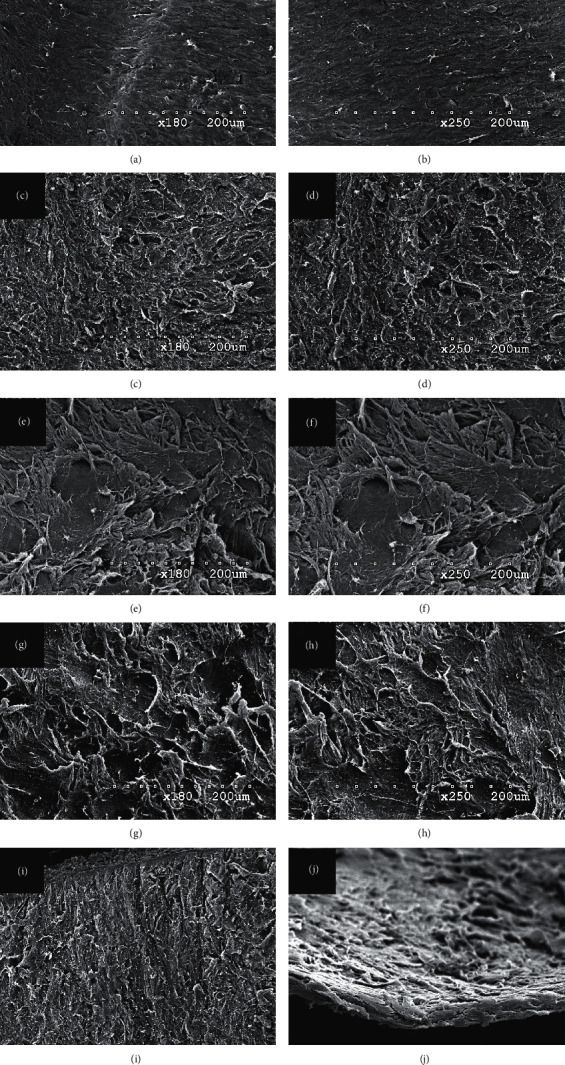
Scanning electron microscopy images of the lenticule surfaces and edges. (a), (b) Anterior surfaces of the lenticules created by Femto LDV Z8 at ×180 and ×250 magnifications, respectively; (c), (d) posterior surfaces of the lenticules created by Femto LDV Z8 at ×180 and ×250 magnifications, respectively; (e), (f) anterior surfaces of the lenticules created by VisuMax at ×180 and ×250 magnifications, respectively; (g), (h) posterior surfaces of the lenticules created by VisuMax at ×180 and ×250 magnifications, respectively; (i), (j) the lenticule edges created by Femto LDV Z8 and VisuMax at ×180 magnifications, respectively.

Table 4 shows the scores of surface roughness and edge irregularity as seen in the SEM images of the lenticules. The mean roughness scores of the posterior lenticule surface in the Femto LDV Z8 group are 3.2 ± 1.1 at ×180 magnification and 3.3 ± 0.9 at ×250 magnification, which are statistically less than the mean sores (4.8 ± 0.8 at ×180 magnification and 4.6 ± 0.7 at ×250 magnification) in the VisuMax group (*p*=0.006 and *p*=0.007, respectively). SEM images of the anterior lenticule surfaces were statistically smoother in the Femto LDV Z8 group than in the VisuMax group at ×180 magnification (*p*=0.039), but no statistical difference at ×250 magnification (*p*=0.102). Meanwhile, there was no statistical difference in the irregularity score of the lenticule edge between the two groups at ×180 magnification (*p*=0.337).  Figure 2 shows the SEM images of the surfaces and edges of the lenticules created by the two femtosecond laser platforms.

## 4. Discussion

To the best of our knowledge, this is the first published report of utilizing Femto LDV Z8 for SMILE procedures. In contrast with the VisuMax, the Femto LDV Z8 has unique features as follows.

One notable feature of Femto LDV models is the low-energy yet high-frequency nJ-level laser system (see Figure 1). With this kind of laser system, the Femto LDV Z6 as published by Riau et al. [12] was documented to have an advantage of faster wound healing. According to Riau et al. [12], the Femto LDV Z6 also provided less flap adhesion strength as compared with the VisuMax. Although no previous study focused on the interface adhesion of lenticules during SMILE procedures, it was found in our study that operations during lenticule separation using the Femto LDV Z8 were much easier than the VisuMax (Ease scores of 1.7 ± 0.6 in the Femto LDV Z8 group versus 3.7 ± 0.6 in the VisuMax group). We speculated that it was directly related to the energy-frequency paradigm of femtosecond lasers during intrastromal incisions. Generally, several parameters of femtosecond laser platforms, such as pulse energy, frequency, pulse duration, distance between adjacent pulses, and pattern of pulses, could affect the cut geometry and surface quality. Compared with the VisuMax 500 kHz femtosecond laser, the Femto LDV had a higher frequency with lower energy laser pulses, which made the Femto LDV not to rely on the formation of cavitation bubbles. Hence, by placing laser spots directly adjacent to each other, the Femto LDV Z8 created a complete and smooth stromal interface for ease of the lenticule separation.

**Figure 3:**
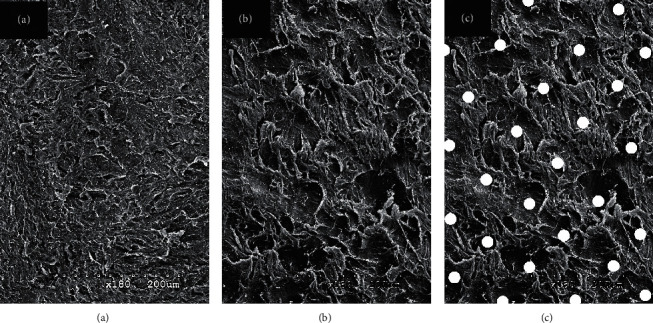
Suspected traces of cavitation bubbles on the lenticule surfaces under scanning electron microscope, ×180 magnification. (a), (b) The posterior lenticule surfaces created by Femto LDV Z8 and VisuMax, respectively. Compared with (a), the rougher surface of (b) was speculated to be caused by cavitation bubbles during VisuMax SMILE surgeries. (c) The white spots were artificially marked on (b) to show the sites of suspected traces of cavitation bubbles.

This speculation could be proven by the roughness performance of the lenticule surface in our study. Although the target incision settings were similar in both laser platforms, smoother surfaces were found on the lenticules created by the Femto LDV Z8 than by the VisuMax (see Table 2 and Figure 2). Because of its low-energy high-frequency platform, the Femto LDV Z8 provided more precise adjacent laser spots as compared with the current commercial laser platforms, which, on the other hand, made the stromal incision cuts similar to that of a microkeratome. However, as mentioned in a previous study [13], the main driving force of intrastromal incision of the VisuMax femtosecond laser was the formation of cavitation bubbles, which looked like a process of tearing a mail stamp and left traces of rough torn surfaces (see Figure 3). Hence, further studies were suggested to evaluate whether this difference in lenticule surface roughness created by these two laser platforms could result in different clinical performances with regards to postoperative optic quality and tissue response [14].

Another special characteristic of the Femto LDV Z8 is its intraoperative OCT and adjustable offsetting. The high-resolution OCT images provided automatic detections of pupil edges, entire structures of cornea layers, and the front surface of lens and iris. In addition, the quantifiable and adjustable offsetting function made it possible for individualized designing the lenticule position without loading and unloading suctions back and forth, which would increase the precision of SMILE procedures and decrease the possibility of surgical complications. On the contrary, because there was no offsetting function in VisuMax, surgeons had to release suction and repeat the docking procedure when the lenticule centration was not satisfied.

As a new SMILE platform, Femto LDV Z8 meant a new learning curve even for the experienced surgeons, as those in our study. Because of the wide range of ease score, the biggest challenge seemed to be in the step of lenticule interface identification (see Table 2). Generally, a “double ring” was visible, signifying the circumference of the cap cut (outer ring) and lenticule cut (inner ring) after VisuMax application. According to the current surgeons' observations, a less conspicuous “double ring” was created by Femto LDV Z8. [15] In our opinion, it was because there were less lamellar bubbles created by the Femto LDV than by the VisuMax. In our study, “two cap opening incisions” strategy was used during identifying lenticules (see Figure 1), wherein the first incision was used to separate the anterior lamellar plane, while the second incision was used to separate the posterior lamellar plane. According to the technique counselor of the Femto LDV Z8, this strategy would decrease the intraoperative complication “unintended posterior plane dissection” [16]. However, in this study, one surgeon (Dr. L) still felt difficulty when he identified the lenticular lamellar planes with an ease score of 6. In the authors' opinion, the safest way for avoiding the “unintended posterior plane dissection” is identifying a doubtless “meniscus sign” [17] using the ledges of the anterior and posterior lamellar planes before separating them.

Like its older generations, the Femto LDV Z8 uses a flat contact glass on its docking system which directly gets in contact with the cornea to minimize lenticule distortion and avoid suction loss during laser applications. Previous studies have proven that the flat suction cone in Femto LDV models induced a significantly higher IOP than the curved suction cone of VisuMax. [18, 19] In Williams et al.'s study [20], the peak IOP generated by the Femto LDV during corneal refractive surgeries could reach up to 201.9 mmHg, compared with the peak IOP of 83 mmHg by VisuMax. Meanwhile, according to the current results and previous studies [20, 21], the Femto LDV needed more time to reach and keep the vacuum (high IOP situation). In the current study, Femto LDV Z8 spent more suction time and more surgical steps than VisuMax (see Table 2). Although the total suction time in Femto LDV Z8 was not evaluated in the current study, it could be easily deduced that the total suction time of Femto LDV Z8 was more than 78.9 s, the time sum of four steps including docking and suction to peak pressure (8.3 s), OCT scanning (12.6 s), offsetting (7.0 s), and laser application (51.0 s). It must be bigger than 35 s, the total suction time of VisuMax mentioned in a previous study [22]. Several complications of refractive surgeries might be due to this high IOP situation, such as optic neuropathy [23, 24], visual field loss [25], and cilioretinal artery occlusion [26]. Although none of these complications was reported to be found in SMILE surgeries or in Fs-Lasik using Femto LDV platforms, we believed that VisuMax would perform lower rate of these complications than Femto LDV Z8 because of the low suction vacuum level during SMILE.

There are several limitations in this study because this is the first experience in SMILE using Femto LDV Z8. First, SMILE was performed on porcine eyes rather than human cadaver eyes. Because of the lack of human cadaver eyes, porcine cornea was thought as a close model for human cornea and was widely used in previous studies. Second is the number of SMILE cases performed in the two groups, although significant differences had been found between two laser platforms. Finally, as an uncommercial model, Femto LDV Z8 has not been accessible in clinical practice. Hence, the current findings would be helpful for improving this laser platform to a better situation.

In conclusion, Femto LDV Z8 showed promising performances as a novel SMILE equipment and would be a hopeful choice for the correction of myopia. It has special and unique features for SMILE procedures, which needs refractive surgeons a slightly new learning curve. With its low-energy high-frequency nJ-level laser system, the Femto LDV Z8 provided smoother surface of lenticule than VisuMax. Further studies are suggested to evaluate the clinical influence caused by the difference in lenticule surface roughness created by these two laser platforms.

## Figures and Tables

**Table 1:** Main equipment parameters of the two femtosecond laser platforms.

	Femto LDV Z8	VisuMax
Manufacturer	Ziemer Ophthalmic Systems AG, Port, Switzerland	Carl Zeiss Meditec, Jena, Germany
Wavelength (nm)	1020–1060	1043
Pulse duration (fs)	200–350	220–580
Laser pulse repetition rate (kHz)	>5000	500
Weight (kg)	215	870
Docking method	Floating a mobile hand-piece on eye	Sliding patient bed under a fixed laser head
Contact glass on suction system	Flat	Curve
Interoperative OCT	Yes	No
Automatic detection of pupil	Yes	No
Pupil central offsetting	Yes	No
Real-time video recording	No	Yes

**Table 2:** Satisfaction scores on the surgical processes using two femtosecond laser platforms.

Platform	Femto LDV Z8				VisuMax			
Eye no.	Eye 1	Eye 2	Eye 3	M ± SD	Eye 4	Eye 5	Eye 6	M ± SD
Surgeons	Dr. W	Dr. L	Dr. Z		Dr. W	Dr. L	Dr. Z	
Ease score of cup incision opening	2	2	3	2.3 ± 0.6	1	2	2	1.7 ± 0.6
Ease score of lenticule interface identification	2	6	2	3.3 ± 2.3	3	3	4	3.3 ± 0.6
Ease score of lenticule separation	2	2	1	1.7 ± 0.6	3	4	4	3.7 ± 0.6
Ease score of lenticule removal	2	2	1	1.7 ± 0.6	2	1	1	1.3 ± 0.0

**Table 3:** Treatment durations of the two femtosecond laser platforms.

Platform	Femto LDV Z8				VisuMax			
Eye no.	Eye 1	Eye 2	Eye 3	M ± SD	Eye 4	Eye 5	Eye 6	M ± SD
Surgeons	Dr. W	Dr. L	Dr. Z		Dr. W	Dr. L	Dr. Z	
Time for suction peak pressure (sec)	8.5	8.3	8.2	8.3 ± 0.2	2.0	2.1	2.0	2.0 ± 0.1
Time for OCT scanning (sec)	12.1	13.0	12.8	12.6 ± 0.5	—	—	—	
Time for offsetting (sec)	6.2	9.8	5.0	7.0 ± 2.5	—	—	—	
Time for total laser application (sec)	50.5	52.3	50.2	51.0 ± 1.1	20.7	22.5	21.0	21.4 ± 1.0
Time for total surgery (sec)	263.3	277.2	254.8	265.1 ± 11.3	164.8	175.7	179.5	173.3 ± 7.6

Sec, second; M ± SD, mean ± standard deviation.

**Table 4:** SEM image scoring of the lenticule surface roughness and edge irregularity.

	Femto LDV Z8									M ± SD	VisuMax									M ± SD	*p* value
	Eye 1			Eye 2			Eye 3				Eye 4			Eye 5			Eye 6			
Anterior surface (×180)	2	3	3	3	4	2	2	3	3	2.8 ± 0.7	3	3	4	4	4	3	3	4	3	3.4 ± 0.5	0.039
Anterior surface (×250)	3	4	3	3	4	3	2	3	3	3.1 ± 0.6	3	4	4	4	5	4	3	3	3	3.7 ± 0.7	0.102
Posterior surface (×180)	3	5	4	4	3	2	2	4	2	3.2 ± 1.1	5	6	5	5	5	5	3	5	4	4.8 ± 0.8	0.006
Posterior surface (×250)	4	5	4	3	3	2	3	3	3	3.3 ± 0.9	5	6	5	4	4	4	4	5	4	4.6 ± 0.7	0.007
Edge irregularity (×180)	2	3	3	3	4	3	2	3	2	2.8 ± 0.7	3	4	3	3	4	4	2	3	2	3.1 ± 0.8	0.337

×180, at ×180 magnification; ×250, at ×250 magnification; M ± SD, mean ± standard deviation.

## Data Availability

The necessary data used to support the findings of the current study can be obtained from the corresponding author on request (Mengmeng Wang, e-mail: wangmengmg@163). Because of the commercial confidentiality, some technical data about the current new mode of Femto LDV Z8 may not be available at the moment.
